# Measurement of Elastic Modulus of Collagen Type I Single Fiber

**DOI:** 10.1371/journal.pone.0145711

**Published:** 2016-01-22

**Authors:** Pavel Dutov, Olga Antipova, Sameer Varma, Joseph P. R. O. Orgel, Jay D. Schieber

**Affiliations:** 1 Center For Molecular Study Of Condensed Soft Matter, Illinois Institute of Technology, Chicago, IL, United States of America; 2 Chemical and Biological Engineering Department, Illinois Institute of Technology, Chicago, IL, United States of America; 3 Departments of, Biology, Physics and Biomedical Engineering, Illinois Institute of Technology, Chicago, IL, United States of America; 4 BioCAT, Sector 18, APS/Argonne National Laboratory, 9700 S. Cass Ave. Argonne, IL, United States of America; 5 Department of Cell Biology, Microbiology and Molecular Biology, Department of Physics, University of South Florida, Tampa, FL, United States of America; 6 Department of Physics, Illinois Institute of Technology, Chicago, IL, United States of America; LAAS-CNRS, FRANCE

## Abstract

Collagen fibers are the main components of the extra cellular matrix and the primary contributors to the mechanical properties of tissues. Here we report a novel approach to measure the longitudinal component of the elastic moduli of biological fibers under conditions close to those found *in vivo* and apply it to type I collagen from rat tail tendon. This approach combines optical tweezers, atomic force microscopy, and exploits Euler-Bernoulli elasticity theory for data analysis. This approach also avoids drying for measurements or visualization, since samples are freshly extracted. Importantly, strains are kept below 0.5%, which appear consistent with the linear elastic regime. We find, surprisingly, that the longitudinal elastic modulus of type I collagen cannot be represented by a single quantity but rather is a distribution that is broader than the uncertainty of our experimental technique. The longitudinal component of the single-fiber elastic modulus is between 100 MPa and 360 MPa for samples extracted from different rats and/or different parts of a single tail. Variations are also observed in the fibril-bundle / fibril diameter with an average of 325±40 nm. Since bending forces depend on the diameter to the fourth power, this variation in diameter is important for estimating the range of elastic moduli. The remaining variations in the modulus may be due to differences in composition of the fibril-bundles, or the extent of the proteoglycans constituting fibril-bundles, or that some single fibrils may be of fibril-bundle size.

## Introduction

Collagen is the most abundant protein in vertebrates [1–6]. In humans, collagen accounts for more than a quarter of the dry protein mass. At present twenty eight different types of collagen have been identified. While some collagen types form fibrils, others form networks and membrane associations. These different types of collagen combine with each other in varying ratios along with non-collagenous molecules to form a variety of possible tissue scaffolds, including basal membranes, ligaments, tendons, cornea, skin and blood vessels.

Type I Collagen is the most common type and the main component of the extracellular matrix of animal connective tissue [1–6]. Its basic structural unit, referred to as a monomer, is made up of three parallel polypeptide chains coiled about each other in a right-handed super-helix. These monomers assemble into fibrils, which are cylindrical cross-linked structures. In turn, fibrils associate with each other in the presence of proteoglycans to form fibril bundles, which historically have also been referred to as fibrils (although both may logically be referred to as fibers as we do here). Whereas a triple-helix monomer is 300 nm long and 1.4 nm wide [5–7], collagen fibrils can extend beyond 1 mm in length, at least in the case of rat tails [8]. Literature values corresponding to the diameter of fibrils exhibit an extensive variation, ranging from 30 to 1000 nm [8,9]. However, this variation is presumably a result of a generalized use of the term “fibril” to describe both discrete fibrils and fibril-bundles alike [2,10]. This seemingly innocuous detail may explain differences between mechanical properties of a fibril and a fibril-bundle. Reassessing literature values in the context of this difference between fibrils and fibril-bundles suggests that fibrils generally appear to have diameters of ~45 nm [11], although it is possible to find individual fibrils that grow thicker [8]. These fibrils and fibril bundles associate with other collagen types and non-collagenous molecules to form higher-order structures such as ligaments and tendons. In these tissues, type I collagen plays vital roles in macromolecular organization and cell signaling as well as bestowing them with load-bearing mechanical properties.

A molecular-level understanding of how connective tissue responds to external stress, or how its mechanical properties are sensitive to genomic variation, requires a precise quantitative knowledge of the mechanical properties of individual fibrils and fibril bundles. Here we present a new method to measure the elastic modulus of isolated collagen thin-fibers. This approach combines optical tweezers [12–16] with atomic force microscopy (AFM), and exploits Euler-Bernoulli elasticity theory. It overcomes two challenges in existing techniques. To circumvent the limitation associated with the maximum force that can be generated by optical tweezers, the method utilizes bending deformation rather than axial stretching. Secondly, it employs a sample preparation or reconstitution protocol that permits measurements in conditions closer to those *in vivo*. Current elastic modulus measurements, almost irrespective of the technique, such as X-ray diffraction [17], AFM [18,19], and micro-electro-mechanical systems (MEMS) [20,21], utilize either drying alone or a drying-soaking cycle for sample preparation that results in sample aging. While aging results in thermal denaturation [22], drying may also lead to irreversible changes in fiber structure [23,24].

Our results show that the longitudinal elastic modulus of type I collagen cannot be represented by a single quantity but rather is a distribution that is broader than the uncertainty of our experimental technique. The longitudinal component of the single fibers elastic modulus is between 100 MPa and 360 MPa for samples extracted from different rats and/or different parts of a single tail. Variations are also observed in the fibril-bundle / fibril diameter with an average of 325±40 nm. Since bending forces depend on the diameter to the fourth power, this variation in diameter is important for estimating the range of elastic moduli. The remaining variations in the modulus may be due to differences in composition of the fibril-bundles, or the extent of the proteoglycans constituting fibril-bundles, or that some single fibrils may be of fibril-bundle size.

## Materials and Methods

We utilize the thin beam Euler-Bernoulli elasticity theory [25] to obtain the elastic moduli of isolated collagen thin-fibers. This theory relates the elastic modulus *E* of a thin cylindrical beam to its length *L*, radius *R* and its end deflection *H* that results from a force *F* perpendicular to the beam axis:

$$E=\frac{4L^{3}F}{3\pi R^{4}H}.$$ (1)

A detailed derivation of the expression above is provided in the S1 File. The estimation of a fiber’s elastic modulus from this expression requires independent measurements of the force-displacement ratio *F*/*H*, the fiber’s length *L* and the fiber’s radius *R*. In addition, it requires that the following three conditions are satisfied: (i) the fiber is a cantilever beam, i.e., it is rigidly fixed at one end while the other end is free; (ii) the fiber has cylindrical shape with constant radius; (iii) the net deflection introduced in the fiber is much smaller than the fiber length.

We obtain *F*/*H* and the fiber’s length from optical tweezers experiments and obtain the radius separately from AFM experiments. The average value of the Young’s modulus is obtained by comparing statistics obtained from these two separate experiments.

Below we provide a brief description of these two methods and also discuss how we check whether the aforementioned three conditions related to the Euler-Bernoulli theory are satisfied.

### Optical tweezers experiments

#### Instrument

**Fig 1:**
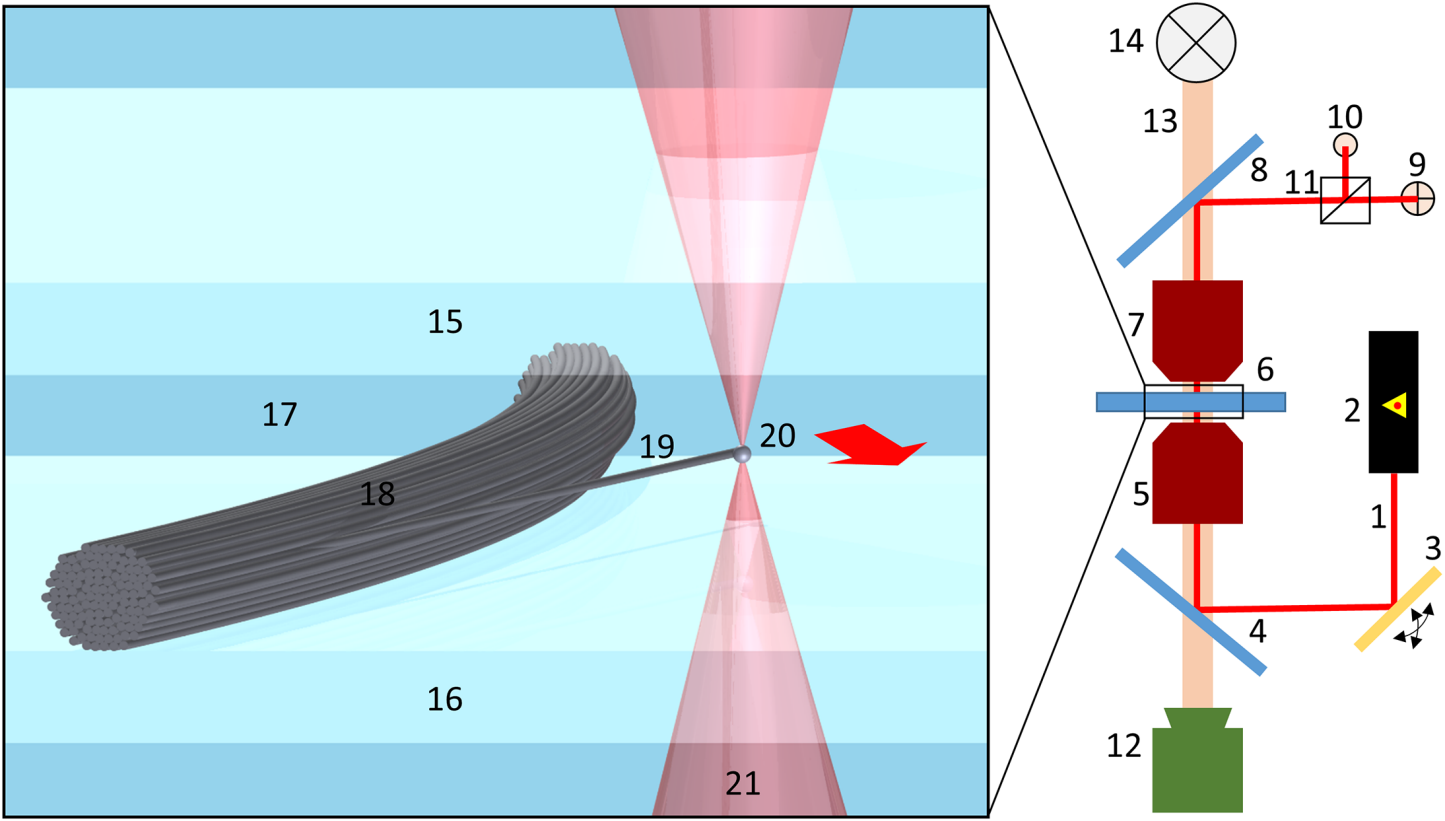
Experimental setup. 1064-nm laser beam 1 is generated by Nd:YAG laser 2, steered by galvo-mirrors 3 and directed by dichroic mirrors 4 into trapping objective 5. Trapping objective focuses laser beam inside sample chamber 6 to create optical trap. After passing through sample chamber, laser beam is collected by tracking objective 7 and projected by dichroic mirror 8 on GaAs quadrant photodiode 9 (for XY detection) and photodiode 10 (for Z detection) after splitting by splitter-cube 11. Experiments are visualized with CCD camera 12 with visible light 13 being generated by overhead LED 14. Top and bottom of the sample chamber are made of glass coverslips 15 and 16 with TBS buffer 17 in between. Collagen fiber bundle 18 with cantilever-like fiber 19 is placed on the bottom coverslip. Microsphere 20 is attached to the cantilever-like fiber with the optical trap 21 and then displaced perpendicularly to the fiber (in the direction shown by red arrow).

For all our experiments we use a commercially available optical tweezers setup NanoTracker ^™^ (JPK Instruments, Berlin, Germany) [26]. The setup is based on a 3W 1064-nm laser, split into two traps by polarization. Precise control over trap position and motion is achieved by use of galvo-mirrors to move each laser beam in the focal plane of objectives plane (Fig 1). We use two 63x confocal objectives (Zeiss C-Apochromat, magnification 63x, numerical aperture 1.2, water immersion, Carl Zeiss Microscopy, LLC, United States) simultaneously. The bottom objective is used for focusing the laser beam to create an optical trap and also for viewing, and the top objective is used to collect the laser beam and focus it on the Quadrant PhotoDiode (QPD) [26].

### Sample preparation

Type I collagen is extracted from rat-tail tendons. Tails were harvested from euthanized control animals used for cardiac research [27,28]. Tails stored at -40°C and thawed for 1 hour at room temperature before preparation. After sample preparation, remaining tail was refrozen. After extracting 2–3 samples, each tail was discarded. Tendon extracted from the tail was immediately immersed in Tris Buffered Saline (Sigma-Aldrich Co., St. Louis, MO). A small portion of tendon cut, perturbed and spread with a needle, and placed in between two glass coverslips in TBS. The gap between the coverslips was nominally 100 μm. Coverslips sealed with acrylic sealant at the edges to prevent evaporation and to comply with the NanoTracker design.

### Force-displacement ratio

Following sample placement in the NanoTracker, the first challenge is to locate a single fiber that satisfies the cantilever beam criterion, that is, the fiber has one end integrated rigidly with the bundle and other end is free. Note that we do not rely solely on visual inspection. As we describe in detail below, any non-cantilever force-displacement behavior is also evident in data analysis, and such data are discarded.

Upon identification of a cantilever-fiber, we remove the sample from the NanoTracker and inject polystyrene microspheres of diameter 1.034 μm (3K1000 Duke Standard Particle Counter Size Standards, mean diameter 1.034 μm ± 0.015 μm, standard deviation 0.01 μm, index of refraction 1.59 @ 589 nm, Thermo Fisher Scientific, Fermont, CA). Due to the presence of proteoglycans in solution, microspheres bind to coverslips or collagen as soon as they are in contact. The average lifetime of a free floating (Brownian) microsphere in sample is about 1 hour. To avoid the situation where multiple microspheres get into one trap during the experiment, microspheres were injected 3–5 mm away from the working region. The tendency to stick to the glass coverslips prevents microspheres from diffusing to the working region. Using the motorized stage, we move to the ‘pool of microspheres’, trap a microsphere and bring it back to the working region. Prior to attaching the bead to the fiber we calibrate the optical trap [29–31] and verify that there is only one microsphere in the trap and that the back focal plane is properly aligned (see S6 File for more details). We bring the microsphere in contact with the fiber, where it is immediately bound by proteoglycans.

Starting from the equilibrium (zero-stress) position, we move the microsphere in the direction perpendicular to the original axis. This net displacement is kept to within 5% of the fiber’s length, which corresponds to an engineering strain < 0.5%. The rate of displacement is chosen such that the viscous drag force is smaller than the noise level. All QPD signals are saved as a function of the trap displacement. After reaching maximum displacement, the trap returns to the equilibrium point with the same rate. This process is repeated at least five times to check for reproducibility and improve accuracy. The entire procedure is repeated 5–6 times, and each time, the microsphere is placed at a different position along the fiber. All experiments are performed within 3 hours from thawing to prevent any aging effects, such as sample degradation.

To gauge the strength of the bond between a microsphere and a fiber, we attach a microsphere to a big fiber bundle, displace the optical trap and compare the measured signal (microsphere’s displacement from the center of the trap) to trap displacement. Since the microsphere and the trap displacement agreed within measurement uncertainty, we conclude that the interaction between the microsphere and the fiber is sufficiently strong for the extent of displacements introduced. We also examine the strength of the interaction between the fiber and the optical trap, and find that the interaction is weak. See Figure K in S5 File for details.

The displacement of the microsphere from its original position $\vec{d}$ is determined by using the trap sensitivity (*S*_*x*_,*S*_*y*_) (in m/V) [29], QPD signals (*V*_*x*_,*V*_*y*_) and trap displacement $\vec{r}$ as

$$d_{j}=r_{j}-\left(V_{j}\left(r_{j}\right)-V_{j}\left(0\right)\right)s_{j},\;j=x,y$$ (2)

**Fig 2:**
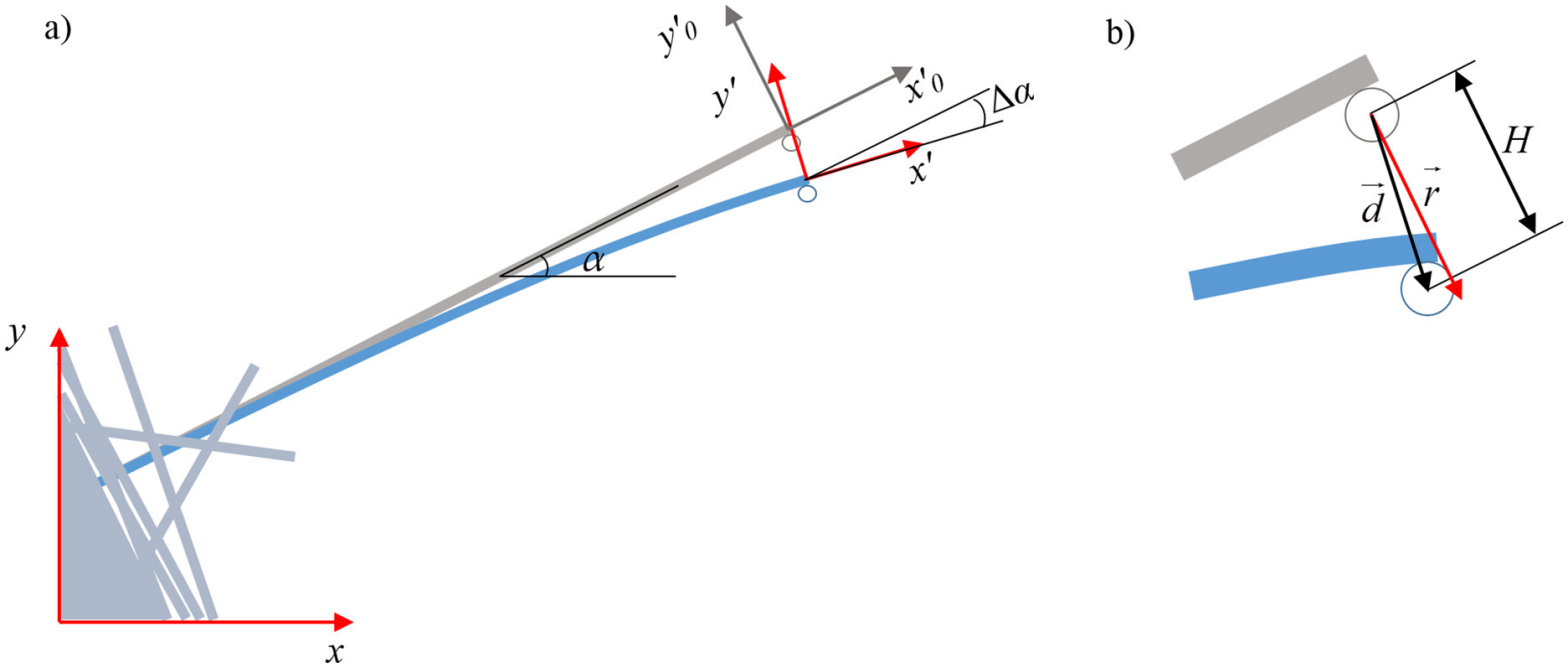
a) Coordinate frames: NanoTracker coordinate frame *xy*, tip of the fiber frame *x*'*y*'. Note that the frame *x*'*y*' is moving with the fiber. The original position (before trap was displacement) of the frame *x*'*y*' is marked *x*'_0_*y*'_0_. Rotation angle between *xy* and *x*'_0_*y*'_0_ is *α*, between *x*'_0_*y*'_0_ and *x*'*y*' is Δ*α*. b) The fiber deflection *H* is a projection of the bead displacement vector $\vec{d}$ on the trap displacement vector $\vec{r}$. Vectors $\vec{d}$ and $\vec{r}$ are not equal due to finite trap stiffness and a force, acting on the bead.

The trap displacement vector $\vec{r}$ is chosen to be perpendicular to the original axis. Consequently, the projection of microsphere displacement vector on trap displacement vector is equal to fiber deflection $H=\vec{d}\cdot \vec{r}/\left|\vec{r}\right|$ (Fig 2b).

We note that when the trap is displaced perpendicular to the fiber’s axis, the fiber can also undergo stretching. However, the strength of optical trap is not sufficient to create any measurable stretching of the fiber. Instead, it is likely that the microsphere gets displaced from the center of the trap in a direction parallel to the fiber. Nevertheless, we note that such displacements are small (< 50 nm), and the trap is still in the linear regime and the bending force can be extracted with good precision as long as we know the instantaneous angle of the fiber Δ*α* (Fig 2a). The latter can be calculated from the known fiber shape *h*(*x*) (See Equation C in S1 File for derivation of the fiber shape)

$$\Delta \alpha ={\text{tan}}^{-1}\left({\frac{\partial h\left(x\right)}{\partial x}|}_{x=L}\right)={\text{tan}}^{-1}\left(\frac{3}{2}\frac{H}{L}\right)$$ (3)

In the coordinate frame *x*'*y*' (Fig 2a), the perpendicular force $F\equiv F_{y\prime }$ is the component of the force vector, acting on the fiber

$$\left(\begin{array}{c} F_{x\prime }\\ F_{y\prime } \end{array}\right)=Q\left(\alpha -\Delta \alpha \right)\cdot \left(\begin{array}{c} V_{x}s_{x}k_{x}\\ V_{y}s_{y}k_{y} \end{array}\right)$$ (4)

where (*k*_*x*_,*k*_*y*_) is trap stiffness (in N/m) [29] and *Q* is the rotation matrix:

$$Q\left(\theta \right)=\left(\begin{array}{cc} \text{cos}\left(\theta \right) & \text{sin}\left(\theta \right)\\ -\text{sin}\left(\theta \right) & \text{cos}\left(\theta \right) \end{array}\right)$$ (5)

### Fiber length

**Fig 3:**
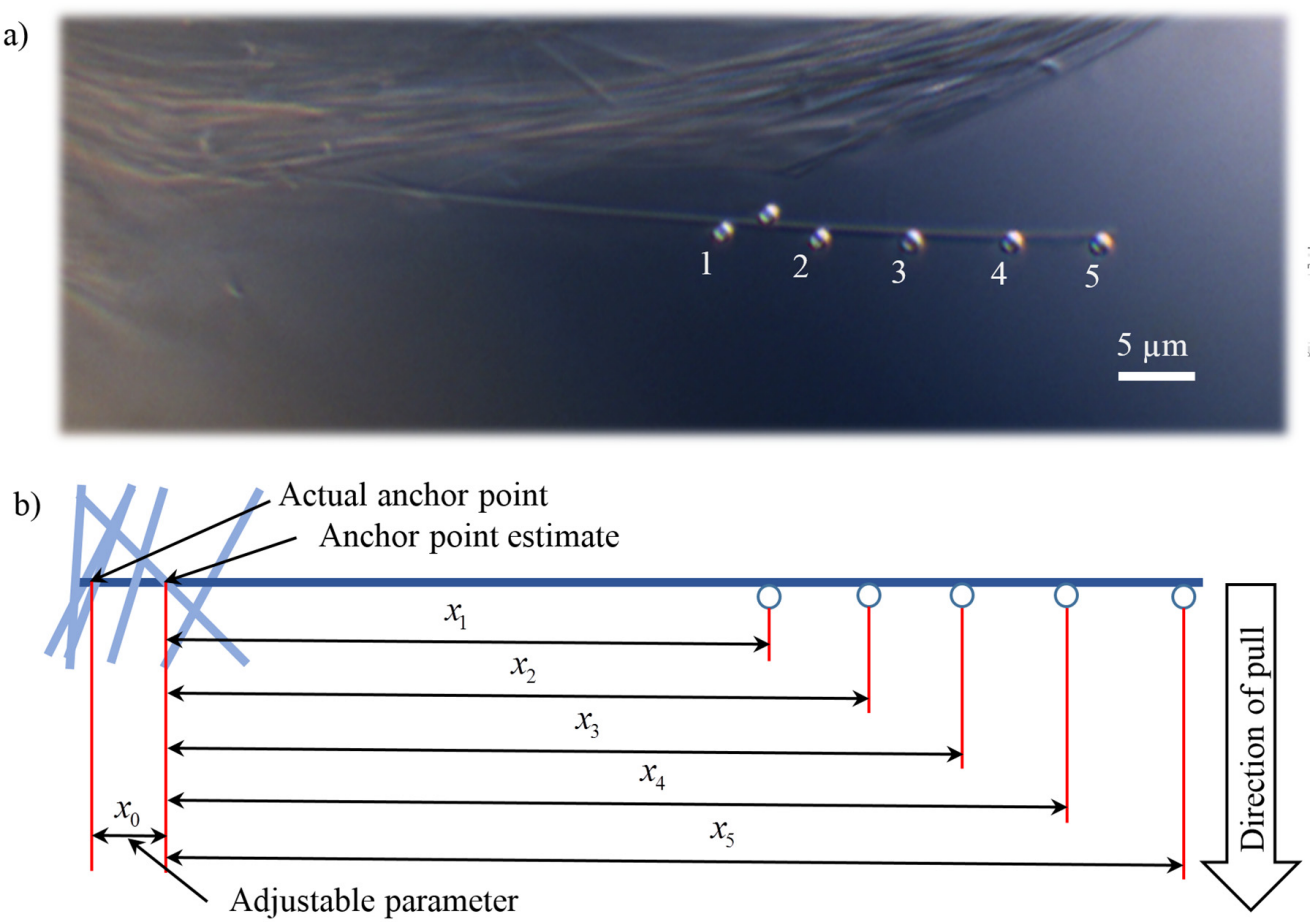
Estimation of anchor point. a) CCD image. The fiber was bent at different positions using microspheres 1–5. Precise estimation of the fiber length *L*_*i*_ for each experiment *i* from just the CCD image is problematic because the region of anchor point is usually blurry. b) Anchor point estimate. The fiber length *L*_*i*_ can be represented as a sum of distance between the bead and initial anchor point estimate (*x*_*i*_) and distance between anchor point estimate and actual anchor point (*x*_*0*_). As *x*_*0*_ is the same for all experiments *i*, it could be precisely estimated during the data processing.

The estimation of the length of the fiber *L* requires knowledge of the position of the anchor, that is, the position where the fiber is attached to the bundle. Since this cannot be discerned unambiguously from CCD images, we obtain it by bending the fiber at different positions along the length of the fiber (Fig 3a). Consider *x*_0_ to be some arbitrary distance between our first estimate of the anchor point and the actual anchor point. The length of the fiber for all other *i* = 1,2,… measurements can then be written as

$$L_{i}=L_{i}\left(x_{0}\right)=x_{i}+x_{0}.$$ (6)

Inserting this into Eq (1) and rearranging yields

$${\left(\frac{H_{i}}{F_{i}}\right)}^{1/3}={\left(\frac{4}{3\pi ER^{4}}\right)}^{1/3}\left(x_{i}+x_{0}\right)$$ (7)

A linear fit yields the slope of (*H*/*F*)^1/3^ as a function of *x* (note that we only using the slope and discard the intersect)

$$B={\left(\frac{4}{3\pi ER^{4}}\right)}^{1/3}.$$ (8)

which can be used to estimate the Young’s modulus,

$$E=\frac{4}{3\pi B^{3}}\frac{1}{R^{4}}$$ (9)

The linearity of the relationship given by Eq (7) also serves as a stringent check for whether the fiber can be modeled as a cantilever beam. In cases where the linearity is not obeyed, such as in cases where the assumed anchor point is loose, the data can be discarded. The use of Eq (9) for estimating the fiber’s Young’s modulus also requires knowledge of the fiber’s radius. We obtain the radius from AFM measurements, as described next.

### Atomic force microscopy

#### Fiber radius

We utilize the AFM manufactured by Asylum Research MFP-3D, Asylum Research, Santa Barbara, CA. Fibers are scanned in TBS buffer using AFM’s tapping mode. The main challenge in AFM scanning is to obtain the appropriate balance between oscillation amplitude, set point and controller parameters—this is necessary for obtaining the best spatial resolution without causing the AFM tip to introduce appreciable deformations in the fiber. Scans are performed with Olympus TR400PSA (spring constant 0.02 N/m). We controlled for spatial resolution by making sure we resolve the D-period of the fiber and for non-penetration of the fiber by comparing the height-to-width ratio of the scanned profile to the one calculated theoretically for a given AFM tip geometry (see S3 File for derivation of height-to-width ratio).

## Results

To estimate the Young's modulus of collagen fibers using the modified form of the Euler-Bernoulli relationship Eq (9), we need to estimate [i] the relationship between the force-displacement ratio and the length of the fiber Eq (7), and [ii] the radius of the fiber. We estimate the former using optical-tweezers experiments, and the latter using AFM experiments.

### Relationship between force-displacement ratio and the length of the fiber

In the bending experiments illustrated in Figs 2 and 3, we displace the microsphere by a distance $\left|\vec{r}\right|=1-2\mu m$ perpendicular to the fiber axis. The rate of displacement is 0.1 *μm*/*s*, such that the viscous drag force is smaller than the noise level. The QPD signals (*V*_*x*_,*V*_*y*_) are recorded at a frequency of 30 Hz. After each bending experiment, the trap is returned to its original position. Overlapping of signals during trap movement and retraction (see Figure J in S5 File) is evidence that the anchor point did not move and that the fiber was not damaged. The bending experiment is repeated five times to check reproducibility. Note that both *V*_*x*_ and *V*_*y*_ contains alignment offsets, so the perpendicular force, calculated according to Eq (4) also contains some meaningless offsets.

**Fig 4:**
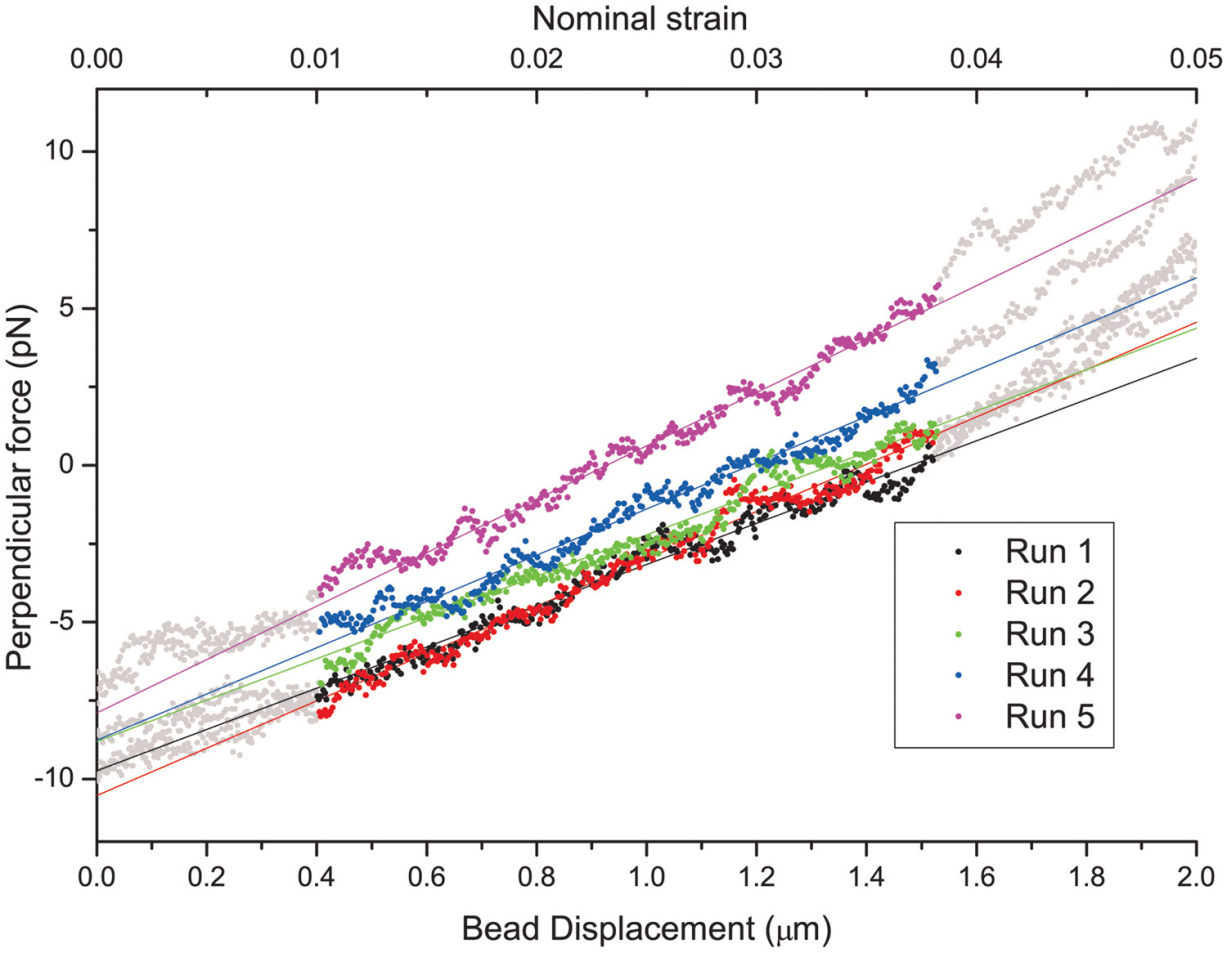
Force-displacement curve and its linear fit. Nominal strain (top axis) is calculated as a relation between fiber length and bead displacement. The fitted region (from 1% to 3–4% nominal strain) is shown in color. We repeat each bending experiment five times (different colors) to improve precession and check reproducibility. The average slope for five runs is 7.32·10^−6^ N/m and standard deviation is 11%. *x*_*i*_ = 28.3 μm.

We fit the linear part of the force-displacement curve (Fig 4) with a linear function using a linear regression method [32]. The linear part usually corresponds to nominal strains up to 3–4%. By ‘nominal strain’ we mean the relation of the displacement to the fiber’s length. We also calculated the engineering strains (see Equation G in S1 File), which happen to be less than 0.5% for all our experiments, which should be sufficient to satisfy the condition (iii). We exclude the region of nominal strain <1% from fitting as some pre-tensioning of the bond between the bead, slight twist of the fiber, or rotation of the bead within the optical trap is possible in that region. During the bending experiments generated bending forces are in the order of 10–50 pN, which corresponds to 20–100 nm of displacement of the bead from the center of the trap. Such displacement is well within the trap linear regime (see Figure I in S5 File for details).

According to Eq (1), the slope of the linear fit is equal to 3*πER*^4^/4*L*^3^. For better precision, slope values are averaged over 5 runs. The intercept corresponds to the alignment offset and is, therefore, meaningless.

**Fig 5:**
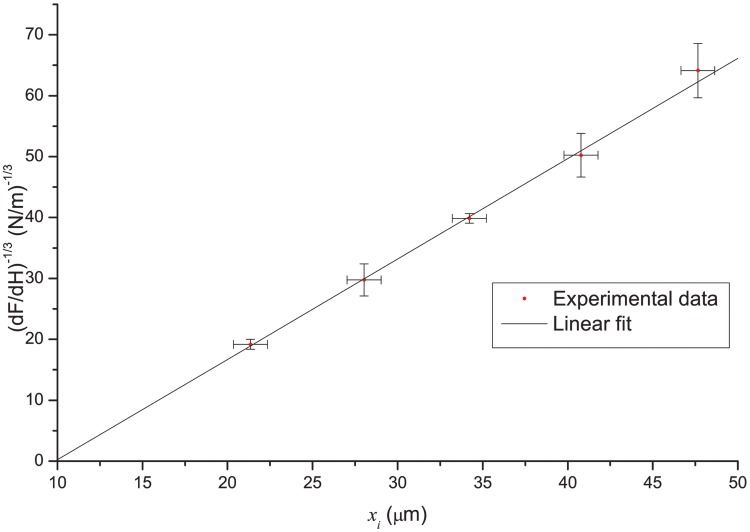
Anchor point determination method. Linear regression allows us to determine both *ER*^4^ and *x*_0_ from fitting parameters. The resulting slope is *B* = 1.65·10^6^ N^-1/3^m^-2/3^ and the intercept is *x*_0_ = − 9.86 μm.

In order to determine the anchor point, we repeat the same experiment with 5–6 beads, attached to different points of the fiber (Fig 3). By fitting the experimental data with a linear function (Fig 5) we determine the slope (Eq (8)) and *x*_0_. The linear dependence on Fig 5 is a strong check for condition (i) as Eq (7) is only linear in case of cantilever beam. Please refer to S4 File for additional check of (i) by fitting the CCD image of bent fiber to the theoretical shape of cantilever beam (Equation C in S1 File).

We performed rigorous error propagation for all experimental steps, starting with uncertainties in the measured forces, positions and displacements, and propagating them through our linear fitting using a linear regression error propagation method [32] (See S7 File for more details). However, uncertainties of *B* and *R* (see the next section on radius measurements) do not converge to a single value of *E*, but rather a distribution which we explain in the Discussion section.

### Fiber Radius Measurement

**Fig 6:**
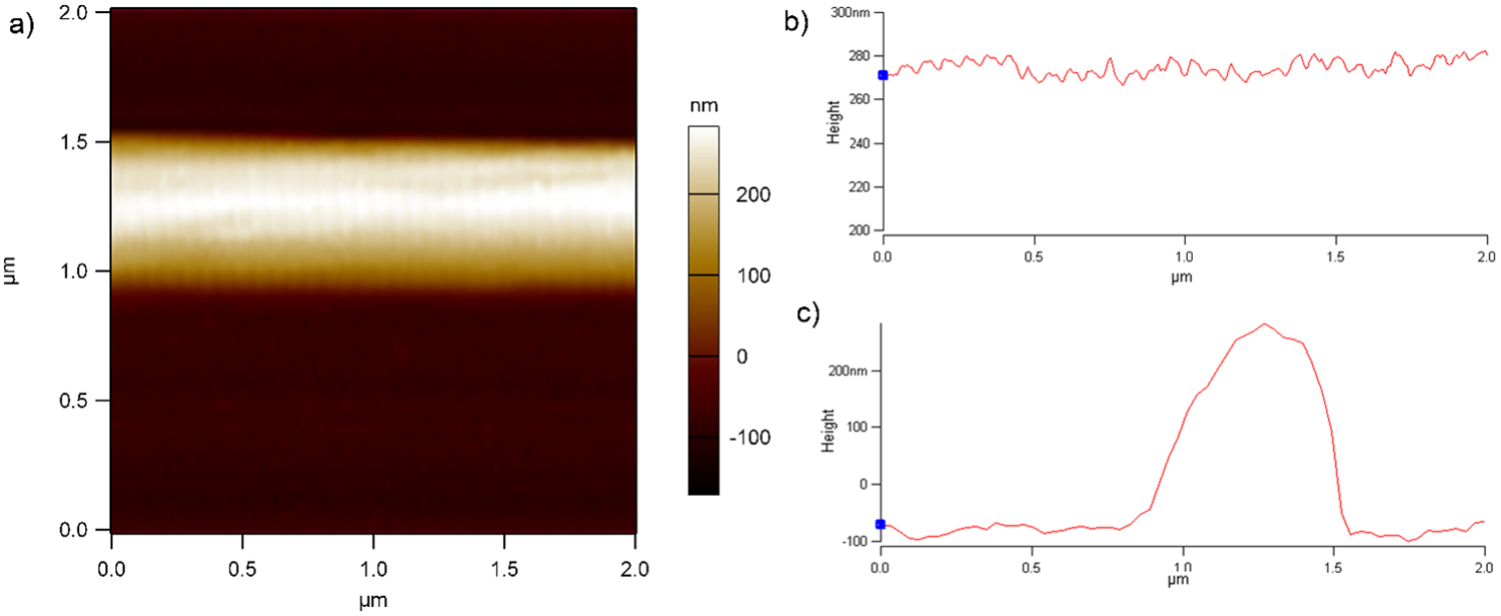
AFM image of single fiber. a) Fiber scan; b) section along the fiber (note the visibility of the D-period); c) section across the fiber.

**Fig 7:**
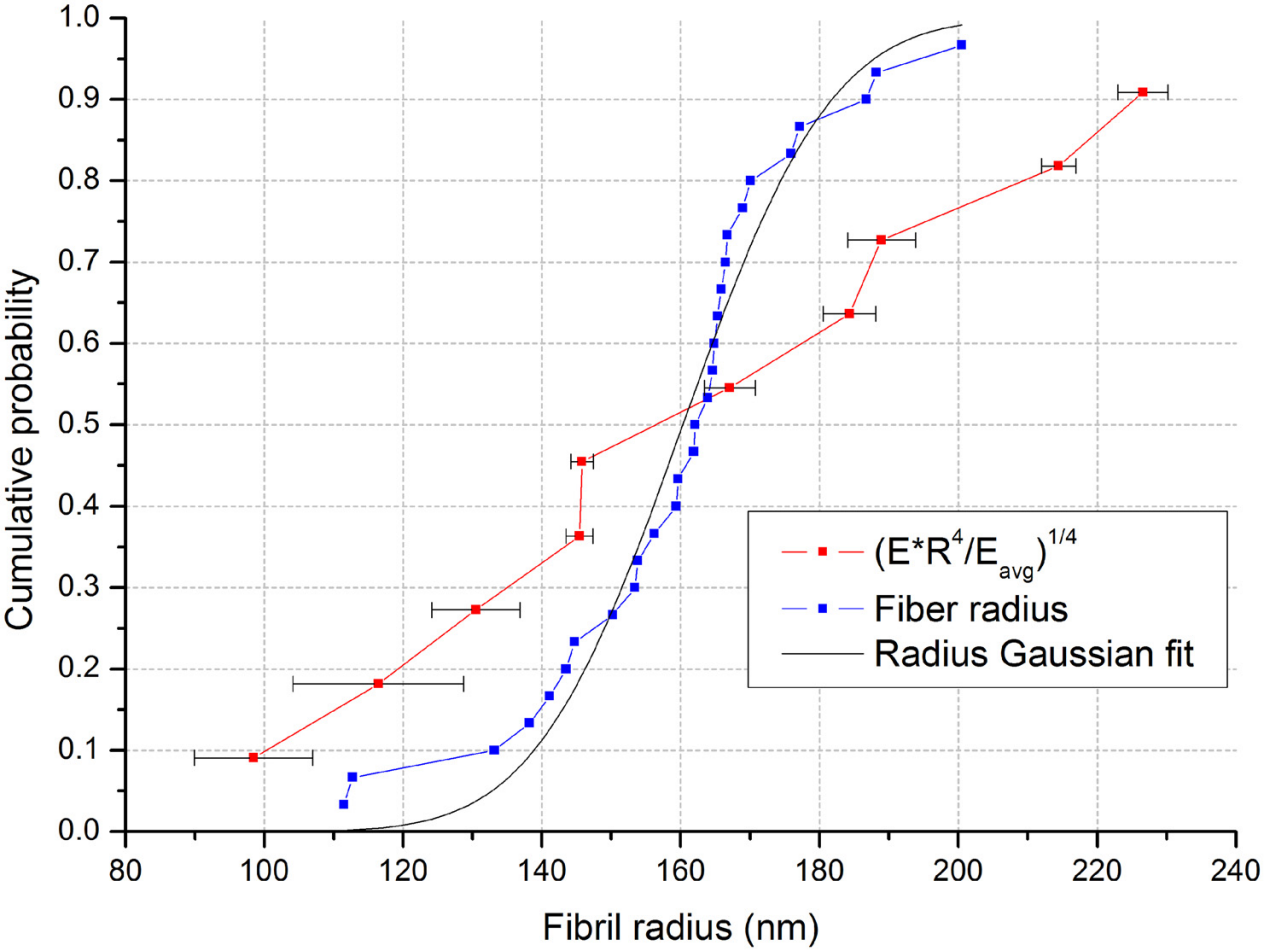
Cumulative distribution of fiber radius and the quantity (*ER*^*4*^/*E*_*avg*_)^*1/4*^. To plot the measured distribution of *ER*^*4*^ together with the fiber radius distribution we divide it by the parameter *E*_*avg*_ and take a quartic root. The value of *E*_*avg*_ that corresponds to the best match between two plots is our best estimate of the average elastic modulus.

To investigate the impact of variance in *R*^4^, we measured the fiber radius distribution by using an ensemble of 29 single fibers. During the scans we used free oscillation amplitude ~ 7nm and set point amplitude ~ 3 nm. One can notice the slight skew of the profile scan (Fig 6c), which is an artifact caused by the shape of the AFM tip holder. Our measurements show that the distribution of fiber radius has shape close to Gaussian with mean of 162 nm and standard deviation 20 nm (Fig 7). The measured fiber D-period of 67 ± 1 nm (Fig 6b) is in agreement with x-ray diffraction results [7]. To measure the radius profile along the length of fiber, we scan a 75 μm portion of fiber and found that the radius varies by less than 5% on that scale, which is also consistent with x-ray scattering [5] results. See S2 File for details.

## Discussion

Using a bending method, we were able to measure directly the distribution of the quantity *ER*^4^ for 10 RTCT I fibers, where *E* is the longitudinal component of the elastic modulus and *R* is fiber radius. We independently measured the distribution of fiber radii using AFM to find a mean of 162 nm and standard deviation 20 nm. To estimate the average value of the longitudinal elastic modulus of RTCT I we plotted the cumulative distribution of quantity (*ER*^4^/*E*_*avg*_)^1/4^ and the cumulative distribution of the RTCT I fiber radius on the same plot (Fig 7), where *E*_*avg*_ is adjusted to give the best match between the two plots. However, to satisfy all 10 measurements of *ER*^4^, *E*_*avg*_ has to be varied between 100 MPa and 360 MPa. This interval is consistent with values reported by other researchers (400 ± 200 MPa, AFM [18], 470 ± 410 MPa, MEMS [20], 123 ± 46 MPa, MEMS [21]), nevertheless, our method allows determination of *ER*^4^ value for each individual fiber with average precision of 7%.

Since the two distributions in Fig 7 have different widths, not all variance in experimental data is accounted for by radius variation. Evidently, there are other sources of variation in the modulus. The elastic modulus could be affected by the proteoglycan density, distribution and composition of their glucose aminonglycan (GAG) chains for the fibril-bundles and the differences between the modulus of a single-fibril and fibril-bundles. According to [7], the fibril is not simple a cylindrical rod, but a tube of type I collagen molecules which is either hollow, or more likely, packed around a FACIT collagen core. However, due to the high-order dependence of the second moment of inertia on the fiber radius, the greater contribution to the bending modulus comes from the outer layer of the fiber, making the detail of the core of lesser significance to this question. The maximum engineering strain (see Equation G in S1 File for derivation) in the fiber appears to be less than 0.5%. All reported experiments were performed within 3 hours from thawing the frozen rat tail, so we also anticipate some (minimal) sample decomposition effects as this is the same or shorter time frame used to prepare rat tail tendon for more sensitive experiments previously [33]. Finally, it also seems plausible that the elastic modulus of the fiber varies among individuals or with position of the fibril or fibril-bundles along the rat tail length. For example, the variation in the proteoglycan concentration along the tail could give a less-elastic/more-stiff composition at the weight bearing portions of the tail.

## Conclusion

By carefully minimizing what may be a common source of preparation-caused artifact, the drying of fibrillar collagen samples, and by recognizing the hierarchical structure of collagen fibers, we provide a plausible explanation for the wide range in elastic modulus reported for rat tail tendons. Differences in proteoglycan content and prominence in collagen fibers, given their known role in fiber elasticity, is likely. Of more significance is a clear indication of measurable differences between what are likely fibrils vs. fibril-bundles (as judged by fiber diameter distributions) in a sample where no distinction has been made previously in studies of this nature. This explanation is also plausible, since structure denotes function and the structure of a fibril vs. a fibril-bundle, while possessing common elements, is not the same.

## Supporting Information

S1 File Derivation of cantilever beam bending force. (PDF)

S2 File Check for the consistency of the radius along the fiber. (PDF)

S3 File Details on fiber radius measurement with AFM. (PDF)

S4 File Fitting the shape of bent fiber. (PDF)

S5 File Optical and viscous error sources. (PDF)

S6 File Experimental protocol details. (PDF)

S7 File Propagation of uncertainty. (PDF)
