# Supplementary material for: Measurement of Elastic Modulus of Collagen Type I Single Fiber
Source: PLoS One. 2016 Jan 22;11(1):e0145711. doi: 10.1371/journal.pone.0145711 (PMC4723153; doi:10.1371/journal.pone.0145711)
Supplement: S1 File — (PDF) [file pone.0145711.s001.pdf]

S1 Derivation of cantilever beam bending force

Given the triple-helical structures making up the fiber, it is reasonable to expect that the fibers have anisotropic elastic properties [31]. However, it has been shown [32] that deflection of a cylinder with anisotropic mechanical properties has the same form as deflection of an isotropic cylinder with the fiber axial elastic modulus being measured instead of an isotropic elastic modulus. For simplicity, we show only the isotropic derivation, however one can refer to [32] for a more detailed analysis.

Thin beam theory, originally developed by Leonard Euler and Daniel Bernoulli, [24] predicts that the deflection $h(x)$ of a thin beam with arbitrary distributed load $q(x)$ [*force/length*] is governed by

$$\frac{d^2}{dx^2}\left(E(x)I(x)\frac{d^2h(x)}{dx^2}\right)=q(x), \qquad \text{Eq. A}$$

where E is the elastic modulus, I is the second moment of inertia, and x is the coordinate along the beam.

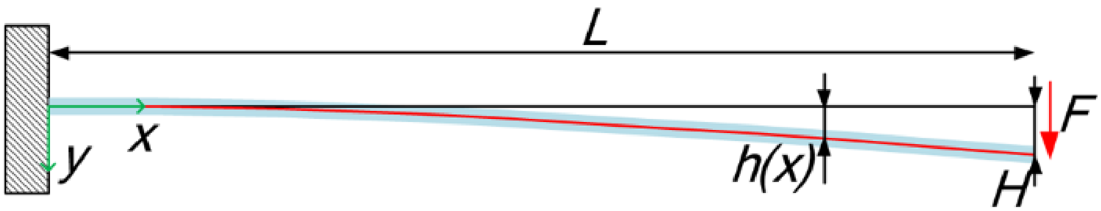

Figure A. Cantilever beam

For a cantilever beam (Figure A) with force F, acting on the end of the fiber (so $q(x)=2F\delta(x-L)$, where $\delta(x-L)$ is the Dirac delta function), E and I independent of x, and with boundary conditions

$$h(0)=0; \quad \left.\frac{dh(x)}{dx}\right|_{x=0}=0; \quad \left.\frac{d^2h(x)}{dx^2}\right|_{x=L}=0 \ , \qquad \text{Eq. B}$$

Eq. A can be solved to find

$$h(x)=\frac{F}{6EI}\left(3Lx^2-x^3\right) \ . \qquad \text{Eq. C}$$

where L is the cantilever length, and F is the force acting on the cantilever's free end.

The second moment of inertia I for a cylindrical rod is [33]

$$I=\frac{\pi R^4}{4} \ . \qquad \text{Eq. D}$$

Combining Eq. C and Eq. D, we find the deflection of the cantilever's end

$$H := h(L)=\frac{4FL^3}{3\pi ER^4} \ . \qquad \text{Eq. E}$$

To estimate the actual (engineering) strain that is applied to the fiber during the bending process we use the following steps. According to [34], the highest strain $\varepsilon_{out}(x)$ of the fiber's perpendicular cross-section made at the position x, occurs in the outer layer of the fiber and can be calculated as $\varepsilon_{out}(x)=\pm\kappa(x)R$, where $\kappa(x)$ is the curvature of the fiber at the position x. Knowing the shape of the fiber Eq. C, we express the curvature as

$$\kappa(x)=\frac{h''(x)}{\left(1+h'^2(x)\right)^{3/2}} \ . \qquad \text{Eq. F}$$

Using Eq. F we find the fiber maximum strain $\varepsilon_{\max}=\max\left(\varepsilon_{out}(x)\right)$ as

$$\varepsilon_{\max}=\frac{4FL}{\pi ER^3} \qquad \text{Eq. G}$$
