# Supplementary material for: Measurement of Elastic Modulus of Collagen Type I Single Fiber
Source: PLoS One. 2016 Jan 22;11(1):e0145711. doi: 10.1371/journal.pone.0145711 (PMC4723153; doi:10.1371/journal.pone.0145711)
Supplement: S2 File — (PDF) [file pone.0145711.s002.pdf]

S2 Check for the consistency of the radius along the fiber

To ensure that the radius of the fiber does not change along its length, we scanned a long (75 µm) section of a dried fiber attached to a glass substrate (Figure B). Small objects surrounding the fiber are crystals of dried buffer.

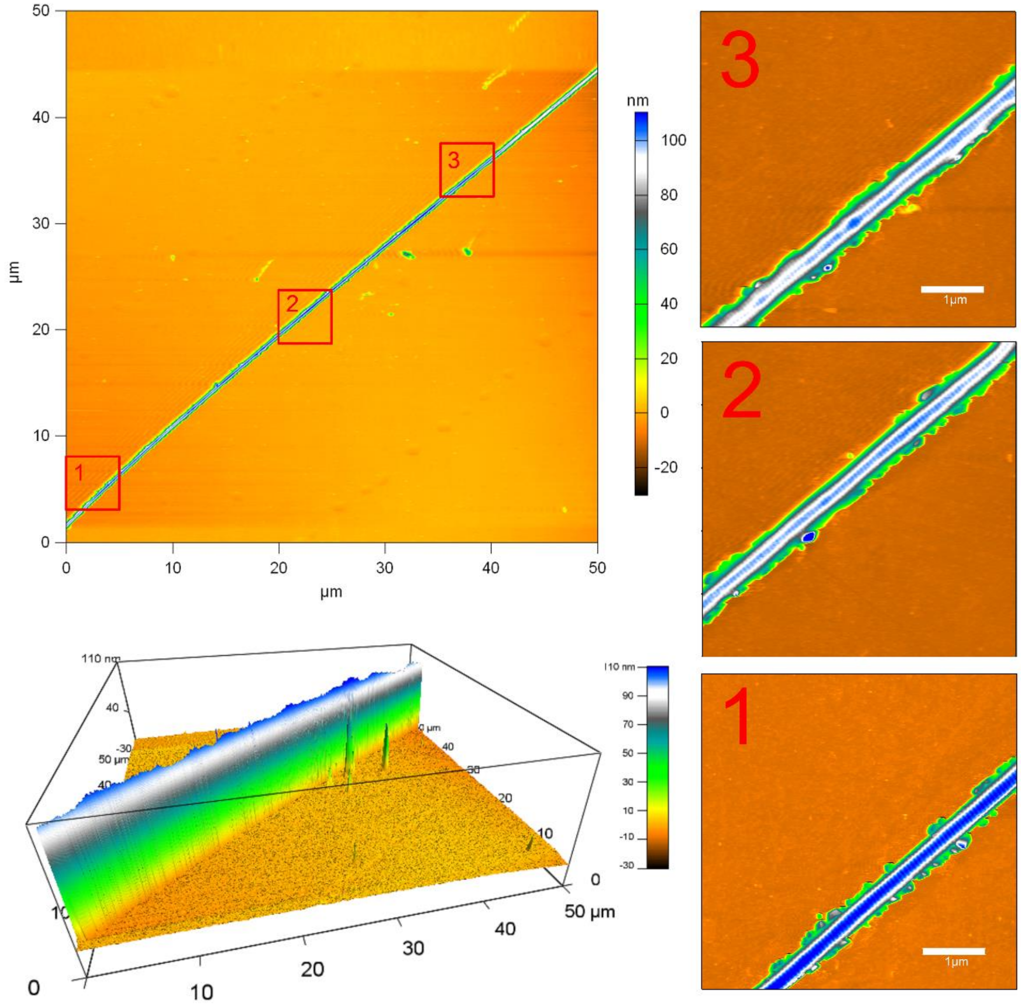

Figure B AFM scan of 75µm section of fiber.

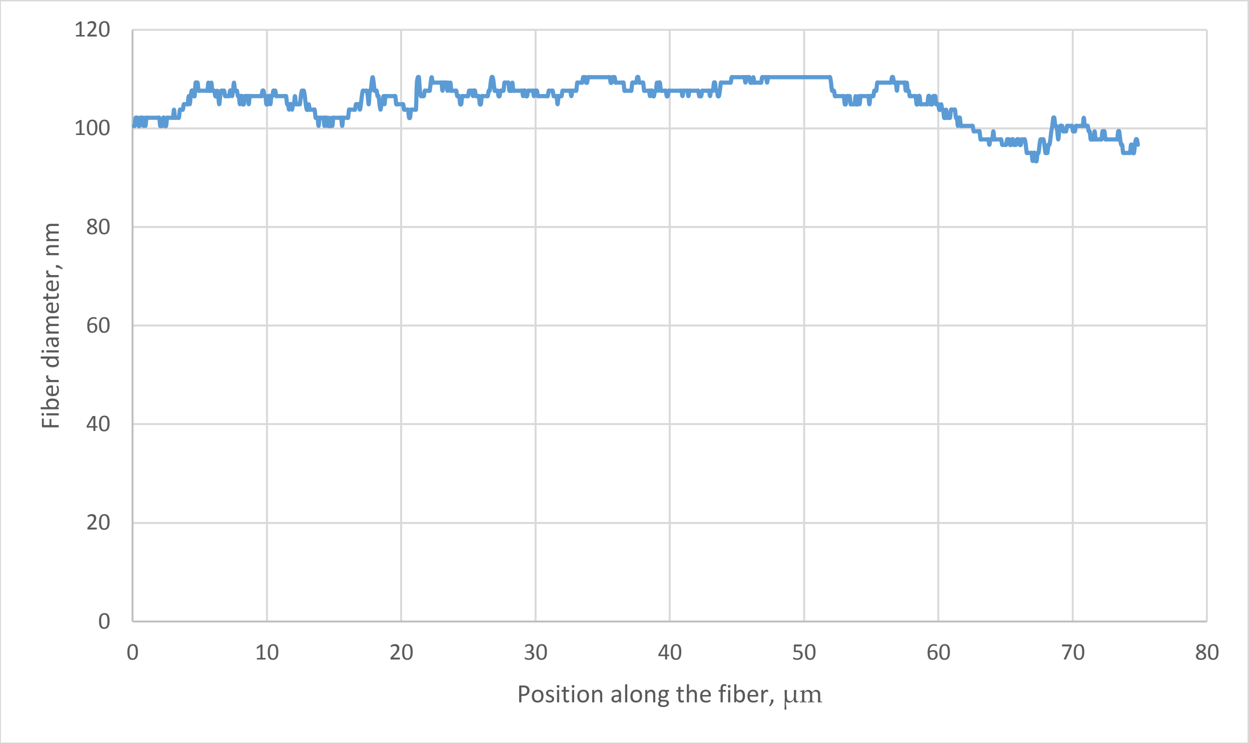

Figure C Diameter of the fiber.

The projection of the maximum height on the XZ plane is shown on Figure C. The fiber diameter is 105.4±4.2 nm.
