# Supplementary material for: Measurement of Elastic Modulus of Collagen Type I Single Fiber
Source: PLoS One. 2016 Jan 22;11(1):e0145711. doi: 10.1371/journal.pone.0145711 (PMC4723153; doi:10.1371/journal.pone.0145711)
Supplement: S3 File — (PDF) [file pone.0145711.s003.pdf]

S3 Details on fiber radius measurement with AFM

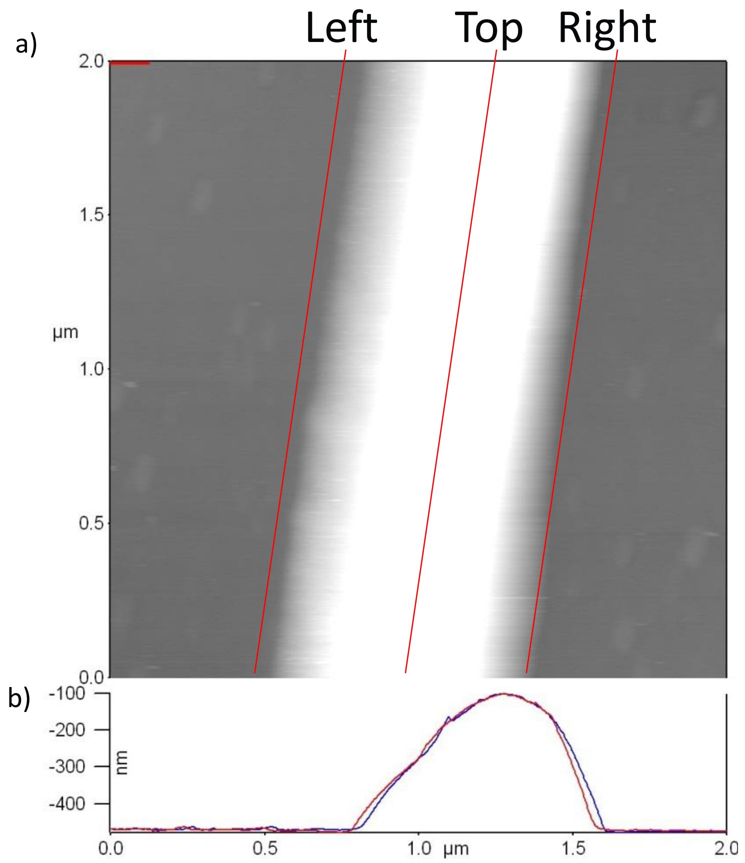

Figure D AFM scan of a fiber. a): 'Left', 'Top' and 'Right' are cross-sections used for calculation of the mean diameter. D-period is not visible only due to scale. b): Cross-section profile. The red and blue lines correspond to Height Trace and Height Retrace, respectively.

To calculate the diameter of the fiber we use the following formula:

$$\text{Fibril diameter} = \overline{\text{Top Height}(l) - \frac{\text{Left Height}(l) + \text{Right Height}(l)}{2}} \qquad \text{Eq. H}$$

Here l is the coordinate along the fiber.

Additional way of checking the AFM settings is performing a deconvolution of the profile shape using the geometry of AFM tip (Figure E).

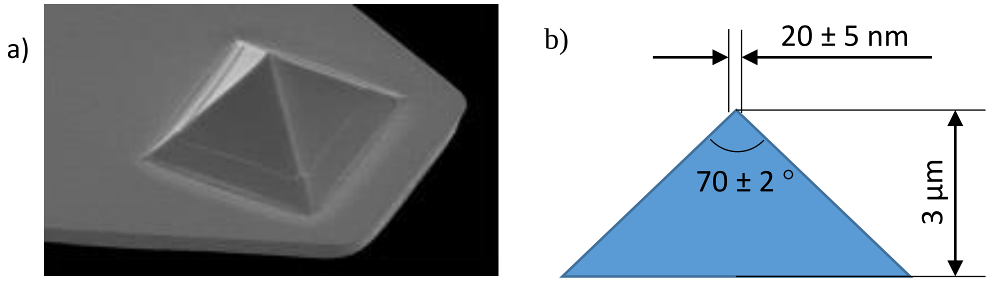

Figure E Olympus TR400PSA AFM tip geometry (a) and dimensions (b). (Data from http://www.asylumresearch.com/ProbeStore)

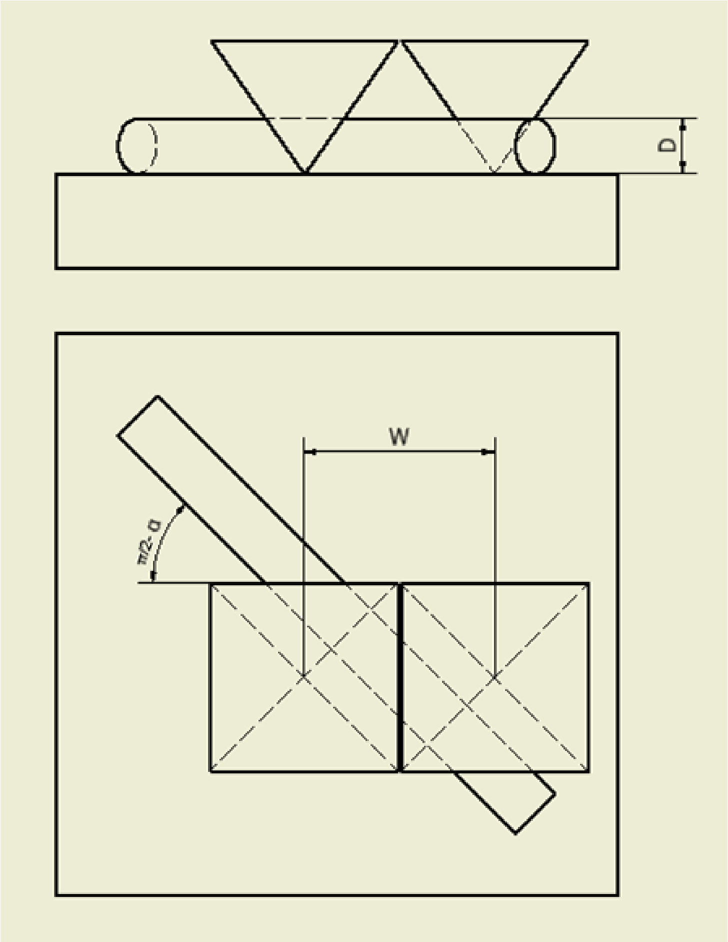

Figure F To AFM scan profile deconvolution

Based on simple geometrical model we derived the theoretical dependence of a relation between width (H) and height (D) of the cross-section profile (Figure D, b) on the angle between the fiber and horizontal axis α :

$$\frac{W}{D} = \frac{1}{\cos\alpha} \frac{1}{\tan\left(\frac{(\pi/2) - \tan^{-1}\left(\tan(7\pi/35)\cdot(\sin\alpha + \cos\alpha)\right)}{2}\right)} \qquad \text{Eq. I}$$

For vertical fiber ($\alpha = 0$) the ratio is $W/D = 1.921$.
