# Supplementary material for: Measurement of Elastic Modulus of Collagen Type I Single Fiber
Source: PLoS One. 2016 Jan 22;11(1):e0145711. doi: 10.1371/journal.pone.0145711 (PMC4723153; doi:10.1371/journal.pone.0145711)
Supplement: S4 File — (PDF) [file pone.0145711.s004.pdf]

S4 Fitting the shape of bent fiber

In order to double check conditions (i) and (ii) we superimpose CCD images of relaxed and bent fibers (Figure) and then measured the fiber's displacement as a function of the position along the fiber.

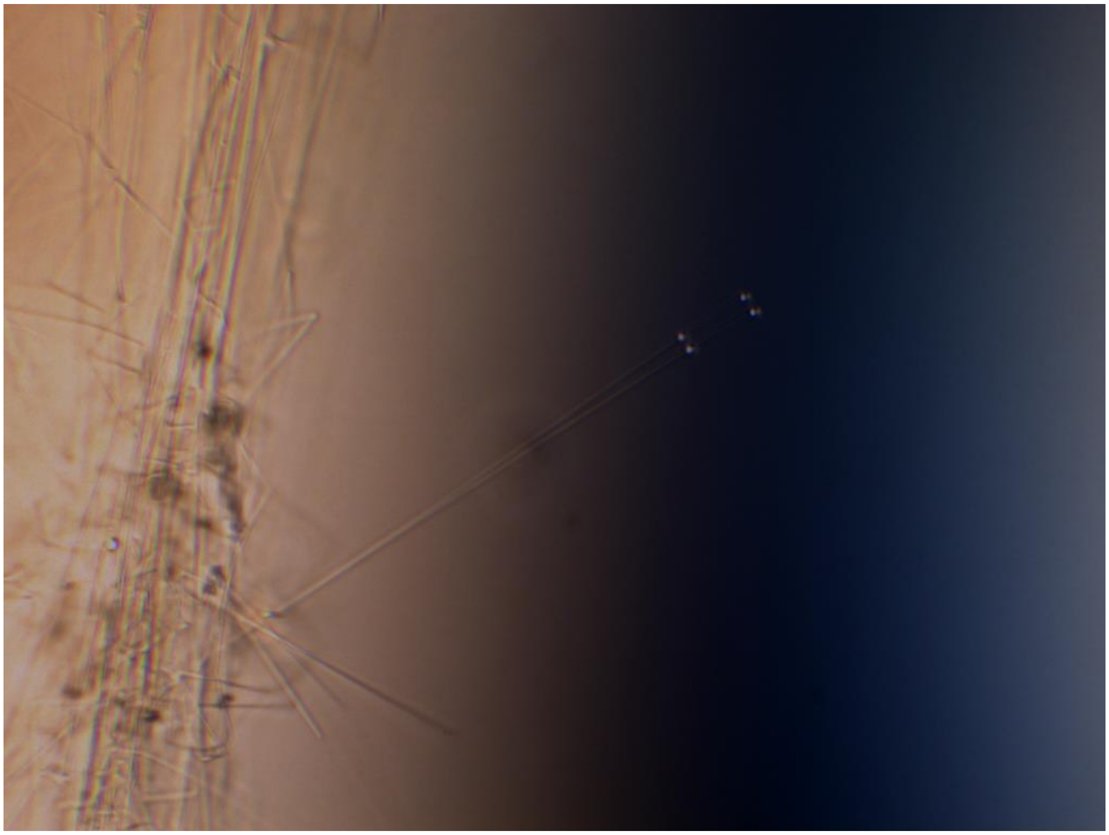

Figure G Superimposed CCD images of bent and relaxed fiber.

For that, we fit the displacements of fiber with the calculated cantilever beam shape (Eq. C in S1 File) for each bead (Figure).

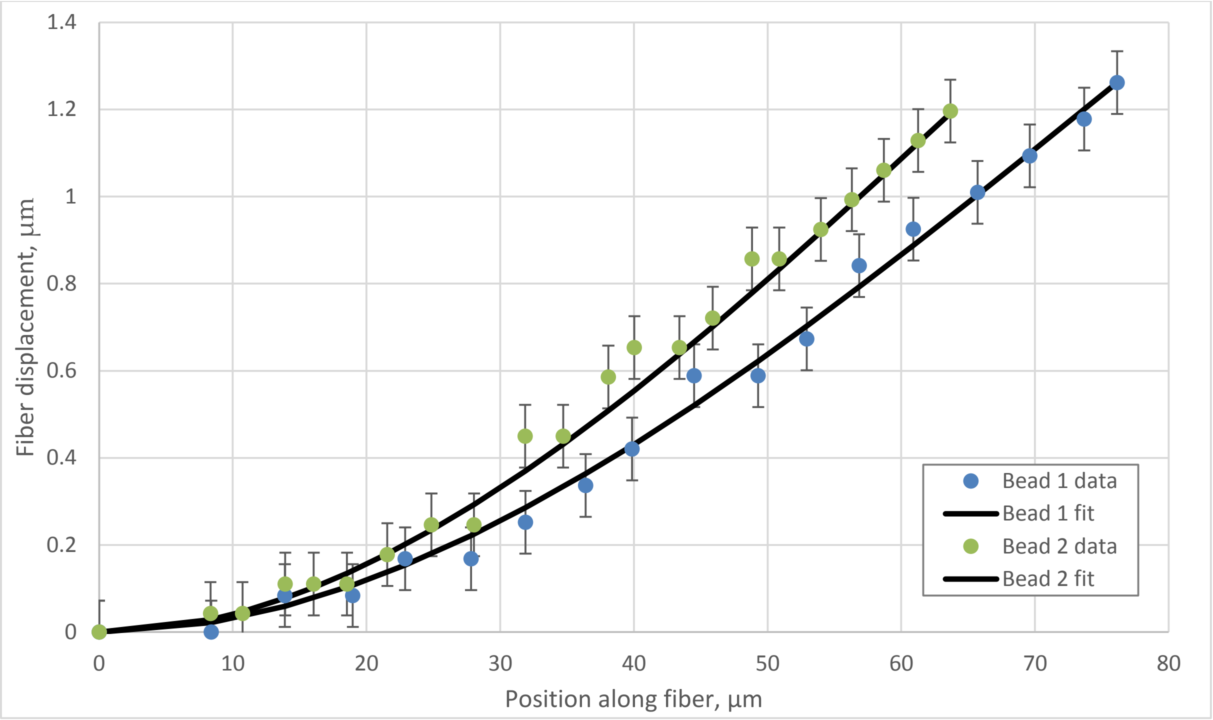

Figure H Fits of the fiber displacements with Euler-Bernoulli equation for cantilever beam. Error bars equal to half of pixel size.
