# Supplementary material for: Measurement of Elastic Modulus of Collagen Type I Single Fiber
Source: PLoS One. 2016 Jan 22;11(1):e0145711. doi: 10.1371/journal.pone.0145711 (PMC4723153; doi:10.1371/journal.pone.0145711)
Supplement: S5 File — (PDF) [file pone.0145711.s005.pdf]

S5 Optical and viscous error sources

Check for the strength of the bond between a bead and fixed fiber.

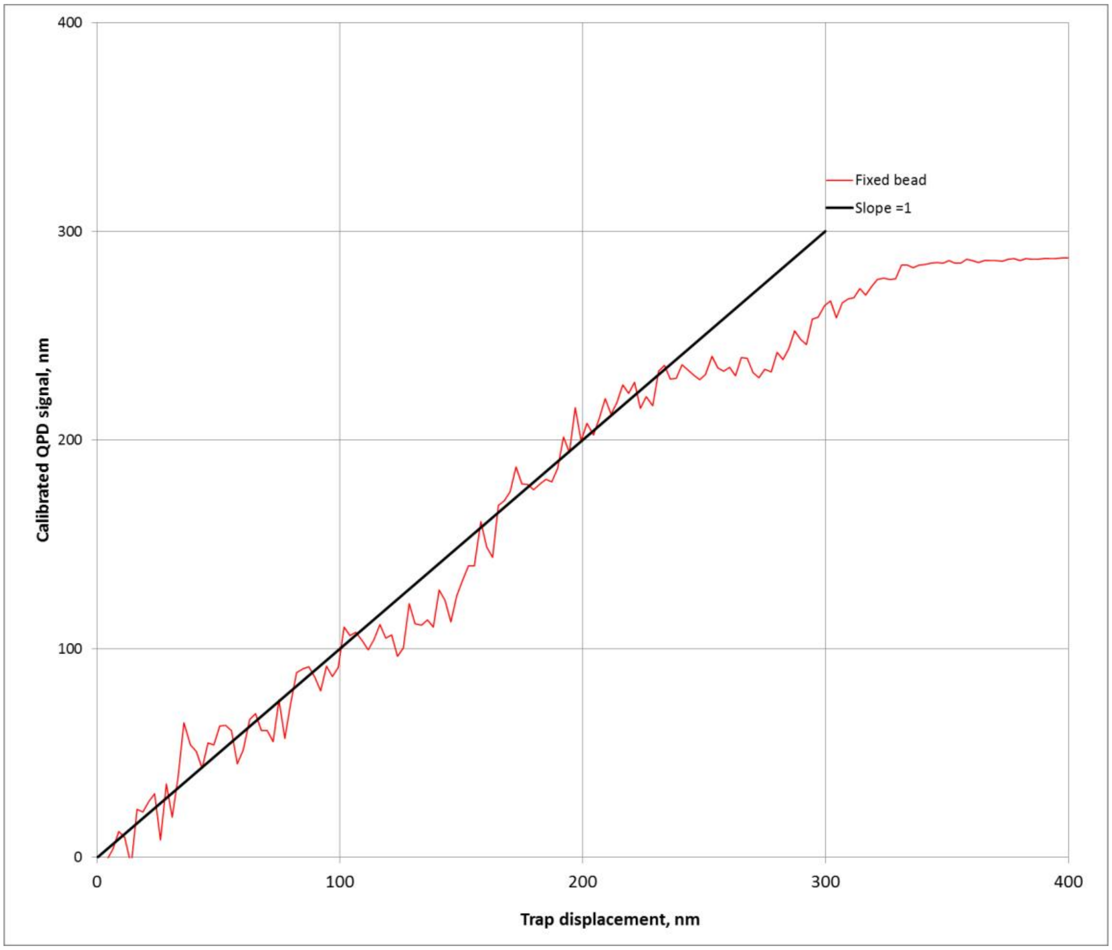

Figure I Comparison of the trap displacement and the measured bead displacement from the center of the trap in the case the bead is attached to a big collagen bundle. The fact that both quantities are equal within uncertainty justifies the strength of the bond. Additionally, one can estimate the linear region of the optical trap as ~±200 nm from the center of the trap.

Stability of sensitivity and stiffness for different beads

**Channel X**								
Bead #	1	2	3	4	5	**Average**	St_dev	St_dev, %
Sensitivity. m/V	3.27E-08	3.12E-08	3.20E-08	3.23E-08	3.17E-08	**3.20E-08**	5.68E-10	1.78
Stiffness, N/m	2.74E-04	2.52E-04	3.06E-04	2.98E-04	3.03E-04	**2.87E-04**	2.30E-05	8.03

**Channel Y**								
Bead #	1	2	3	4	5	**Average**	St_dev	St_dev, %
Sensitivity. m/V	3.33E-08	3.33E-08	3.28E-08	3.31E-08	3.35E-08	**3.32E-08**	2.49E-10	0.75
Stiffness, N/m	4.68E-04	4.23E-04	4.83E-04	4.77E-04	4.52E-04	**4.61E-04**	2.40E-05	5.22

Table A Comparison of sensitivities and stiffnesses for different beads.

Displace-retract comparison

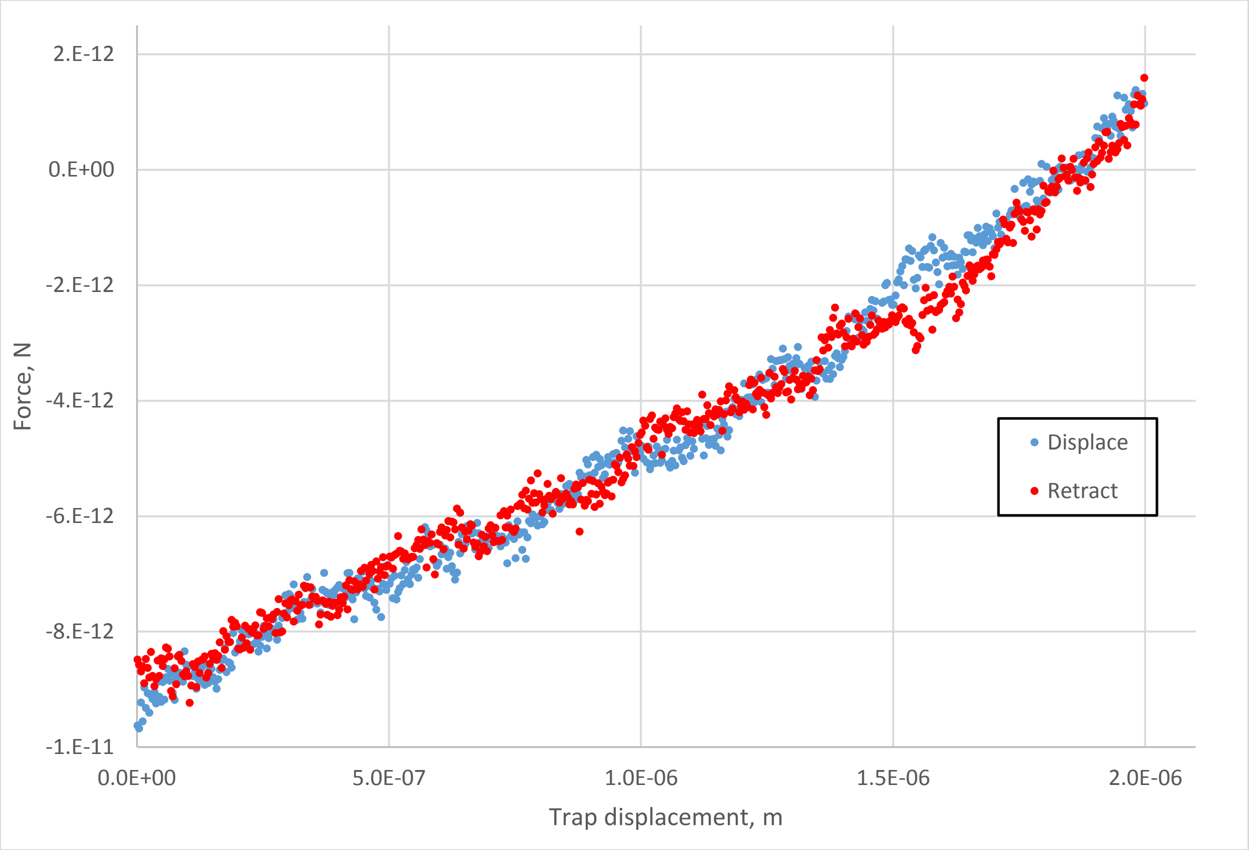

Figure J Comparison of the QPD signal during the bead displacement and retraction. Overlap of the plots shows that the anchor point did not irreversibly moved and that the fiber was not damaged.

Interaction of the fibril with the trap:

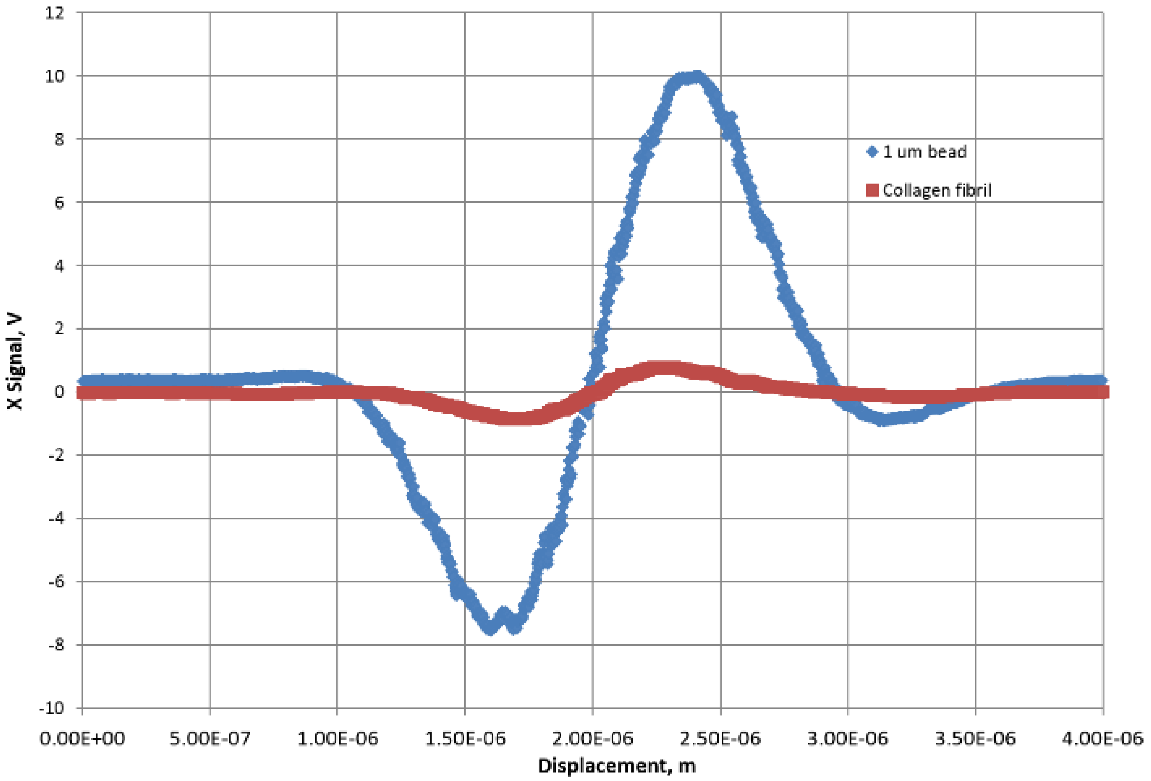

Figure K Comparison of optical trap scans over a bead (diamonds) and a fibril (squares), fixed to the glass. Note that signal distortion from the fibril at the distance $R_{bead} + R \approx 0.7\mu m$ is small comparing to the signal from the bead in the center of the trap (~1÷3%).

Effect of viscous drag:

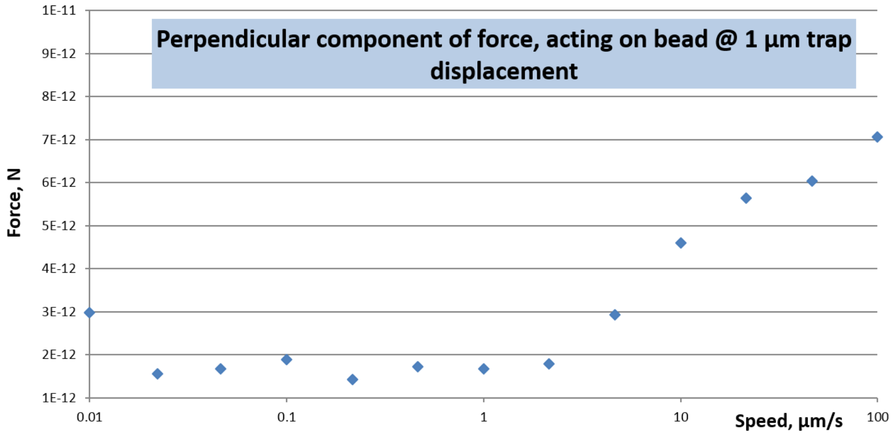

Figure L Dependence of force, acting on the bead on velocity of trap movement. One can notice that at high velocities viscous drag effect is dominating. Therefore, all bending experiments were performed at velocity 0.2µm/s
