# Supplementary material for: Measurement of Elastic Modulus of Collagen Type I Single Fiber
Source: PLoS One. 2016 Jan 22;11(1):e0145711. doi: 10.1371/journal.pone.0145711 (PMC4723153; doi:10.1371/journal.pone.0145711)
Supplement: S6 File — (PDF) [file pone.0145711.s006.pdf]

S6 Experimental protocol details

When we have trapped a bead close to the cantilever-like fibril, we need to go through a few preliminary steps:

- We first ensure that there is only one bead in the trap. Multiple beads align vertically which makes it hard to visually distinguish from a single bead. Therefore, we temporarily switch the trap off and observe the Brownian motion of the bead.

- After retrapping the bead we record the thermal motion of the bead in the trap, calculate its power spectral density and fit it with a Lorentzian shape in order to calibrate the trap, i.e. calculate trap stiffness ("spring constant", in N/m) and detector sensitivity (in m/V).

- Then we move the trap with the bead in it and record the QPD signals V_x and V_y to ensure the stability of the signal in the working region. This step helps ensure the absence of any parasitic signal in that region. Due to the design of the experimental setup, misalignment of focal planes of trapping and tracking objectives could cause slight misalignment of the position of the back focal plane of tracking objective and QPD, which in turn causes interference spikes in the signal while the trap is moved. If spikes are present, we readjust the tracking objective position to eliminate the spikes or move them away from working region.

- When the fiber is parallel to the confocal microscope focal plane, we save a CCD camera screenshot and measure the angle between the fiber and the camera's horizontal axis. In case the fiber is not parallel to the confocal microscope focal plane (we can move the trapping objective up or down to focus on different parts of the fiber), we save screenshots of both the anchor point and the free end, overlap them using graphics processing software and measure the fiber's angle.

- Using the motorized stage, the fiber tip is brought to the working region and the bead is carefully placed on the fiber tip.

Starting from the equilibrium (zero-stress) position, we move the bead in a direction perpendicular to the original fiber angle for 2-3% of the fiber's length. The trap speed was chosen such that the viscous drag force is smaller than the noise level. All signals from the QPD are saved as a function of trap displacement. After reaching maximum displacement, the trap returns to the equilibrium point with the same speed. This experiment is repeated five times to check reproducibility and improve accuracy.

The entire procedure is repeated for 5-6 different beads with each bead placed on different positions along the fiber. This permits checking the condition (i) (that the fiber is a cantilever beam), calculating the position of the anchor point (which is not always easy from observing the CCD camera image) and enhancing the accuracy of Young's modulus estimates.
