# Supplementary material for: Measurement of Elastic Modulus of Collagen Type I Single Fiber
Source: PLoS One. 2016 Jan 22;11(1):e0145711. doi: 10.1371/journal.pone.0145711 (PMC4723153; doi:10.1371/journal.pone.0145711)
Supplement: S7 File — (PDF) [file pone.0145711.s007.pdf]

S7 Propagation of uncertainty

We express uncertainty of the measured elastic modulus ΔE in terms of fundamental uncertainties of our experiments, namely: $\Delta F, \Delta x_i, \Delta R$ and ΔH .

Assuming that these fundamental uncertainties are uncorrelated, we can use the following expression:

$$\Delta f\left(y_1..y_n\right)=\sqrt{\sum_{i=1}^{n}\left(\frac{\partial f}{\partial y_i}\right)^2 y_i^2} \qquad \text{Eq. J}$$

In our case $E=\frac{4}{3\pi R^4 B^3}$, so

$$\Delta E=\sqrt{\left(\frac{\partial E}{\partial B}\Delta B\right)^2+\left(\frac{\partial E}{\partial R}\Delta R\right)^2}=E\sqrt{\left(-\frac{3\Delta B}{B}\right)^2+\left(-\frac{4\Delta R}{R}\right)^2} \quad , \qquad \text{Eq. K}$$

Where

$$B=\frac{d\left(dF_i/dH_i\right)^{-1/3}}{dx_i}. \qquad \text{Eq. L}$$

ΔB (which is the uncertainty in the slope on Fig 5) comes from linear regression error propagation ([29], Chapter 15.3) and based on uncertainties in $\left(dF_i/dH_i\right)^{-1/3}$ and uncertainty Δx_i .

Let us define $A_i := \frac{dF_i}{dH_i}$ and $A_{i,j} := \frac{dF_{i,j}}{dH_{i,j}}$, where i is the number of the bead, j is the number of the run and $A_i = \sum_{n=1}^{j_{\max}} A_{i,n} \Big/ j_{\max}$.

Now

$$\Delta\left(A_i^{-1/3}\right)=\frac{\partial A_i^{-1/3}}{\partial A_i}\Delta A_i=-A_i^{-4/3}\Delta A_i/3 \qquad \text{Eq. M}$$

$$\Delta A_i = \sqrt{\frac{\sum_{j=1}^{5}\left(\Delta A_{i,j}\right)^2}{20}} \qquad \text{Eq. N}$$

Finally, uncertainty in $\Delta A_{i,j}$ is calculated using linear regression based on ΔF and ΔH.

Fundamental uncertainties are equal to:

Δx_i - uncertainty in bead position and consists of CCD camera pixel size and optical aberrations in tracking objective. By using the JPK Optical Calibration Slide it was checked that aberrations are small (undetectable) compared to pixel size.

For that reason Δx_i = ± ½ pixel size = ±70 nm = $\pm 7 \cdot 10^{-8}$ m

ΔH - uncertainty in bead displacement, comes from uncertainty in trap position and uncertainty of bead position relative to the center of the trap. In the described experiments the spatial resolution was reported to be ±5 nm = $\pm 5 \cdot 10^{-9}$ (see JPK Technical Report: Spatial and temporal resolution of optical tweezers). The uncertainty in the trap position is of the same order, so total uncertainty ΔH = ±10 nm = $\pm 10^{-8}$ m

ΔF - uncertainty in measured force. The lower limit of measured force (thermal noise floor) for given trap stiffness is 1.5 pN. Additive to that is parasite signal due to optical aberration, dust on objective and imperfectly adjusted BFP (base line signal) – all together about 1 pN. Also, uncertainties caused by trap calibration (20% of value), interaction with fibril (5% of value) and laser power instability (5% of value) should be taken into account. Drag force was shown to be negligible under experimental conditions.

ΔR - uncertainty in R, measured to be ~ 12%

	Bead position	Bead displacement	Measured force	Fiber radius
Typical value	20-70 μm	0.5-1.5 μm	5-50 pN	160 nm
Systematic uncertainty	-	5 nm	20.6%	1-2%
Measurements per experiment	1	300-500	300-500	29
Total uncertainty	70 nm	10 nm	1.8 pN + 21%	20 nm
Total uncertainty, %	0.1-0.35 %	0.7-2 %	22-42 %	12 %

Table B Comparison of uncertainties for measured quantities. Note that even though uncertainty for measured instant force can be high, only 20.6% of it is systematic and the rest get significantly decreased by repeated measurements, taken during a single experiment.
